# Use of Robotics in Gait Rehabilitation Following Stroke: A Review

**DOI:** 10.7759/cureus.31075

**Published:** 2022-11-04

**Authors:** Vaishnavi Warutkar, Ragini Dadgal, Utkarsha R Mangulkar

**Affiliations:** 1 Department of Neurophysiotherapy, Ravi Nair Physiotherapy College, Datta Meghe Institute of Medical Sciences, Wardha, IND

**Keywords:** robotics, stroke, hemiplegia, gait, exoskeleton

## Abstract

A stroke is an acute disruption of focal or global brain activity that last for a day or leads to death. Most stroke patients have an asymmetric gait, lower-extremity stiffness of the affected (hemiplegia) side, and impaired single stance and weight transfer capacity, restricting their locomotor function. Although between 65% and 85% of individuals can walk alone within six months after a stroke with appropriate surgical/pharmaceutical procedures and rehabilitative therapy, poor walking and cardiac efficiency continue to impede everyday walking for hemiplegia patients. Various methods are used to improve gait in stroke patients. Robotic-assisted gait training (RAGT) is given via a robot system device.

Ground exoskeletons, end-effector devices, wearable exoskeletons are three types of rehabilitation robots that have been developed. The HAL (Hybrid Assistive Limb) exoskeleton and RoboGait is also modified gait device to enhance gait. Robotic Neurorehabilitation can be a useful technique for reducing gait impairments and, as a result, increasing the standard of living of post-stroke individuals.

## Introduction and background

A stroke furthermore referred to as a cerebrovascular event, is a significant disturbance of localized or overall brain function that lasts for a day or results in death and is thought to be caused by a vascular event. Although medical improvements have reduced stroke mortality and morbidity, the effects on stroke survivors and the community remain considerable [1]. As per the WHO (World Health Organization), stroke is the world's second-largest reason for fatality, following cardiovascular illnesses [2,3]. In adults, stroke is the major factor causing a long-time impairment. The occurrence of hemiplegia is growing annually as a result of an aging population and in emergency treatment, resulting in considerable medical and societal consequences [4]. Motor loss is a very well consequence of stroke because it impairs muscle function and movement. Several hemiplegia sufferers have difficulty moving, and one of the major aims of therapy is improving gait. Because the process of spontaneous recovery can be completed possibly in as short as 2-3 months, early rehab is critical for individuals to optimize their functioning following a stroke [1]. Involvement in gait-related activities includes not just walking activity but also taking into account the context and surroundings as it changes when walking, such as uneven terrain, level differences, avoiding obstacles, and crowding [5,6]. Most stroke patients have an asymmetric walking pattern, lower-extremity stiffness on the affected (hemiplegic) side, and impaired single stance and weight transfer capacity, restricting their locomotor function [4]. While the majority of recovery is expected to occur within the first few weeks following hemiplegia, individuals may continue to make progress on functional activities for many months [7,8]. Spasticity is one of the major causes of activity limitations as well as gait and equilibrium issues in individuals with stroke in the acute stages of the disorder [2]. Twenty percent of people remain wheelchair-bound for three months following a stroke, and around 70 percent walk at a decreased speed and ability [9]. Although 65-85 percent of patients may walk independently within six months after a stroke with appropriate [4] surgical/pharmaceutical procedures and rehabilitative therapy. Poor gait and cardiopulmonary endurance continue to impede everyday walking for stroke patients [4].

Robotics' advent in recent times has produced intriguing recovery results for stroke victims, providing an option to conventional physiotherapy [10,11]. Rehabilitation robots are mechanical interactive gadgets that enable limb movement for both sensorimotor and, maybe, cognitive recovery [12,13]. Executive ability and attention management are both parts of cognitive performance when walking [14]. These robots may operate in two or three dimensions based on the overall design. They are made using a variety of functioning mechanisms, including strength training, basic passive mobilization, and robot-assisted mobilization, which impedes in varying degrees with the patient. The majority of robotics allows for interaction in a virtual setting. The technical complexity of these various systems varies quite a bit, referring to the fact that these technologies are still developing [15].

## Review

Robotics in rehabilitation to improve gait

Multiple therapists execute traditional rehabilitation therapy for post-stroke walking training manually. This is time-consuming, ineffective, and also costly [4]. Advanced concepts prefer a task-specific repetitive method [16]. In past times, it has even been demonstrated how more levels of walking training resulted in improved outcomes for persons who have suffered a stroke [9]. Furthermore, therapeutic outcomes are dependent on the individual skills of therapists; therefore, patients do not have access to uniform and standardized therapies. A training session involving at least two therapists is also essential for individuals with lower-limb spasticity. As a result, individual patient training dosages are limited. The progression and evaluation of advanced rehabilitation robots in healthcare settings are critical for bridging this gap in rehab treatments and ensuring training dosages for stroke patients [4].

Gait rehabilitation using robots first emerged two decades ago as an option for manual gait training. Robotic gait rehabilitation, in comparison to conventional treatment, may provide highly regulated, repeated, and rigorous training in an engaging setting, minimize the therapist's physical workload and give objective and quantitative assessments of the individuals' development [17]. Patients with more severe disabilities may gain more from robotic training, regardless of other criteria, except for the requirement for residual trunk control, which has been recognized as a positive predictive factor for robotic walking training [18]. The invention of Lokomat in 1994 brought in the usage of gait rehabilitation robots. Despite these advancements, the best rehabilitation robot for a certain user with a neuromuscular condition is still unknown [17].

Several meta-analyses have recently established the efficacy of several task-oriented retraining strategies for hemiplegia patients, such as body weight-supported treadmill training (BWSTT), circuit class training, enhanced fitness treatment, and mechanized walking training. For the latter case, electromechanical robotic machines are used to automate lower limb motions during locomotion. These systems were created to assist physical therapists by improving the security, speed, and consistency of non-robotic BWSTT, generating intricate multimodal stimulation, providing comprehensive external biofeedback to the individual, and limiting working expenditure [19]. The robotic device also allows partial or full-body weight-bearing supports for non-ambulatory individuals to be recruited and falls to be avoided during training sessions [20].

Treadmill therapy is suitable for hemiplegia patients who are able to walk, and electromechanically-assisted training is especially beneficial for individuals who are not yet able to walk [21,22]. From 1980, robot-assisted gait training (RAGT) had already been utilized to help individuals whose neurological conditions have created motor abnormalities [21,23]. The RAGT is given via a robot system device which is been operated by Lokomat-certified physical therapists. A motor-driven gait exoskeleton was fitted to the individual's lower extremities as part of the lower limb training system. Across a synchronized treadmill, the individual's entire body is stabilized by a bodyweight support structure. The individual's legs were led on the treadmill by a pre-programmed physiological gait pattern, which was conveyed to levers via the bodyweight support system, which induced the stance as well as swing phases. The method provided a precise amount of guidance that was suitable for the patient's clinical state to improve walking speed, strength, as well as quality while decreasing the patient's harmful compensatory gait pattern and unnecessary stress [1]. 

Although these benefits, there is contradictory information regarding the efficacy of RAGT [24]. Robot-assisted gait rehabilitation can lengthen, intensify, and increase the days of treatment sessions, easing therapist workload as well as decreasing treatment expenditures [25].

Robotic technology

Ground exoskeletons (e.g., Lokomat, LOPES, ALEX), End-effector tools (e.g., Gait Trainer, Haptic Walker), and also wearable exoskeletons (e.g., Lokomat, LOPES, ALEX) are three types of rehabilitation robots that have been developed. In addition, "soft exoskeletons" or "Exosuits" that incorporate light actuation mechanisms and/or materials to help the locomotion component have recently been developed [17]. The end effector concept defines that the participant's foot is positioned on footplates whose paths imitate the stance as well as swing stages throughout gait retraining, although exoskeleton systems include programmed motors or passive parts that move the knees as well as hips throughout gait phases [25]. Robotic exoskeletons for gait retraining direct the limbs along physiologic walking patterns that have been modulated, whereas the subject receives proprioceptive feedback that is almost typical after limb loading [26].

Wearable exoskeletons are growing rapidly as a breakthrough technology for gait retraining because of the active engagement needed from the client, which encourages physical exercise, and also the ability to be utilized as community assistive equipment. The number of research on wearing exoskeletons has increased in the last 10 years, following the current trend toward robotic devices. Few of these tools have previously received FDA clearance and/or the CE (Conformite Europeenne) marking and are applicable for purchase, while many are still in the progression stage [17]. Exoskeletons are mechanically and concurrently attached to the human body, with the risk of obstructing and friction with normal limb movement. As a result, various essential biomechanical aspects, including the degrees of freedom (DoF), ranges of movement (ROM), and also joint torque, have been included in the designing of limb exoskeletons [25].

The HAL (Hybrid Assistive Limb) orthosis is a modified gait device with a control structure aimed at capturing the recipient's voluntary activities. Cybernic Voluntary Control (CVC) and Cybernic Autonomous Control (CAC) are two components of HAL that allow for both voluntary and automated action. Each of these systems relies on the intention of the user, but in various ways. HAL is initiated in the CVC mode by the recipient's voluntary lower extremity muscle activation, as measured by surface electromyography, which produces torque and assists gait movements. The CAC mode can be employed if there is no more voluntary action of the walking muscles [27]. The Hybrid Assistive Limb has been demonstrated to be effective for gait conditioning for individuals having lower limb weakness in both subacute and long-term stages following a stroke [28].

RoboGait is an automated locomotor rehabilitation framework that includes a robotic lower-limb orthosis, dynamic weight support that may be adjusted, a synchronized treadmill, and biofeedback tools. The lower limb system is an electric motor-driven orthosis that may be adjusted to fit various patient sizes. The electric motors also power the counterweight system and the patient lift, which may be operated from the remote-control device. The moving pace is adjusted to match the treadmill velocity and changed as needed to avoid scuff marks. A computer oversees the entire system. The device software allows for the storage of extensive patient and session details, as well as therapy reports [2]. Lower extremity recovery robots can repeat Gait patterns on behalf of a physiotherapist for extended durations of time and provide clinical outcomes by aiding and adjusting the person's movement [29,30]. Additionally, robotic assistance may encourage repetitive and intense training in a safe setting [31,32].

Discussion

The objective of the review was to analyze the benefits of robotics in the rehab of hemiplegia patients to improve gait. This article includes a few studies which state the efficacy of robotics to augment locomotion in post-hemiplegia patients.

A study was done by B.P.H. Chung [1], which included a total of 41 participants from that few received conventional physiotherapies while others were given Robot-assisted gait training (RAGT), and it was proposed that RAGT could provide stroke individuals additional advantages regarding ambulation, mobility, and balance. However, the impact of RAGT on stroke individuals is comparable to that of conventional physical therapy in terms of basic ADL.

In research carried out by M. Pohl et al. [16] on 155 non-ambulatory patients from which group A underwent locomotor exercises with physical therapy, and group B received only physiotherapy. This concluded that the participants who underwent locomotor exercises and physiotherapy had considerably enhanced gait function in comparison with the patients who receive physiotherapy alone. An identical study was done by Li et al. [4], who suggested that patients with stroke experienced improved locomotion and lower-limb motor function following training with the suggested exoskeleton robot. However, its outcomes were equivalent to those of traditional training. Robotic therapy is being used in therapeutic practices, but it is not meant to take the position of therapists; rather, it is meant to give individuals more options for secure and efficient practices.

In research done by Erbil et al. [2], 48 hemiplegia individuals received botulinum toxin-A treatment, in which 32 patients were given robot-assisted training while 16 were given conventional physical therapy. It was concluded that in chronic hemiplegia patients whose spasticity was treated with BoNT-A, an integrated management plan, including RAT and physiotherapy, may offer even more advantages. In a study by Kubota et al. [23], patients received a session of 90 minutes with a wearable robot. It was found to be beneficial and safe to perform on patients even during certain periods after the subjects received their diagnosis.

In research done by Cho et al. [33], 20 individuals were divided into either group A, which was given robot-assisted gait training (RAGT) followed by traditional physiotherapy, or group B, which underwent the same therapies in the opposite sequence. And the study found that RAGT improves motor activity and trunk stability along with balance and gait ability. A study by Jayaraman et al. [34] discussed the limitations of various robotic devices which should be taken into consideration. Exoskeletons may be utilized more often in homes and health centers if they are viable. The development of robotic equipment will keep progressing rapidly.

A summary of a few articles is given below in Table 1.

**Table 1:** A summary of a few articles included in the study.

Sr. no.	Author	No. of subjects	Intervention	Conclusion
1.	BP Chung [1].	14-RAGT group and 27-control group	Traditional physiotherapy and robot-assisted gait training (RAGT)	The RAGT group benefits more in terms of ambulation, motor activity, and balancing as compared to another group.
2.	Erbil et al. [2]	32- RAT group and 16- control groups	Robot-assisted training (RAT) and conventional physiotherapy	Management with RAT and physiotherapy provided furthermore impact in chronic hemiplegia patients.
3.	Li et al. [4]	130 individuals were separated into the Robot and Control group.	Conventional physiotherapy, lower-extremity exoskeletal robots were utilized for the locomotor training session	The therapy robot in this research was able to help hemiplegic individuals with their locomotor ability, but its results were no different from those of regular traditional locomotion training.
4.	Mehrholz et al. [9]	62 trials including 2440 individuals.	Training gait with electromechanical assistance in addition to physical therapy and gait training without using any devices	Following hemiplegia, electromechanically aided gait retraining in conjunction with physical therapy seems to be more likely to result in unassisted walking than other approaches.
5.	M. Pohl et al. [16]	155 patients	Group A underwent locomotor exercises and physical therapy; group B received only physical therapy	The capability to walk more easily was considerably improved by intensive locomotor training and physical therapy.
6.	Rodríguez‑Fernández et al. [17]	87 clinical studies	Wearable exoskeletons, physical therapy	Wearable exoskeletons for lower extremities were designed to assist walking in persons with neuromuscular disabilities.
7.	Morone et al. [18]	100 patients	Robotic and conventional gait training	Individuals with more severe disabilities could impact more by robotic training.
8.	Dierick et al. [19]	40 patients	Conventional physical therapy treatment and exoskeleton robotic-assisted gait training (RAGT)	All these groups showed almost identical functional progress and advantages.
9.	Iosa et al. [20]	20 patients	Electromechanically assisted gait training	The findings offer a justification for choosing gait trainer (GT) parameter values.
10.	Bruni et al. [25]	13 randomized control trials	Physical therapy approaches, in addition to robotic devices and Conventional gait training	The findings support the concept that using robots can improve hemiplegic patients' gait rehabilitation outcomes.
11.	Wall et al. [27]	32 patients	HAL (hybrid assistive limb) training, the traditional method of gait training	In terms of any post-intervention outcomes, this study did not find any distinctions among the groups.
12.	Wall et al. [28]	140 participants	HAL (hybrid assistive limb) for gait retraining	When used to retrain patients with lower-limb paresis, the HAL method was found to be more effective.
13.	Cho et al. [33]	20 patients	Traditional physiotherapy, robot-assisted gait training (RAGT)	A study found that robot-assisted gait training is beneficial for enhancing motor function and trunk stability in addition to balance and gait ability.

## Conclusions

Robot-assisted devices should be employed in stroke patients with significant functional disabilities to enhance gait rehabilitation. It will also improve motor function in patients. Robotic support might promote rigorous, repeated training. Several gait training approaches, like a hybrid assistive limb and robot-assisted gait device, described in the study can be applied for additional benefits. Robotic neurorehabilitation can be a helpful technique for reducing gait impairments and, as a result, increasing the quality of life of post-stroke patients.

All these advancements in physiotherapy rehabilitation of a stroke patient should be made available in clinical settings at cost-effective rates for better and faster recovery of the patients. 
